# Conservative Management of the Ring and Little Finger Carpometacarpal Joint Dorsal Dislocations

**DOI:** 10.7759/cureus.48745

**Published:** 2023-11-13

**Authors:** Chijioke K Orji, Charles O Ojo, Oluwatobi O Shekoni, Ajibola A Adebisi

**Affiliations:** 1 Trauma and Orthopaedics, Wrexham Maelor Hospital, Wrexham, GBR; 2 Surgery, United Lincolnshire Hospitals NHS Trust, Grantham, GBR; 3 Surgery, Glan Clwyd Hospital, Bodelwyddan, GBR; 4 Surgery, Peterborough City Hospital, Peterborough, GBR

**Keywords:** emergency department, orthopaedics, plaster of paris, male prisoner, hand injury, conservative management, penthrox, gutter splint, closed reduction, carpometacarpal dislocation

## Abstract

This is a case of right ring and little finger carpometacarpal joint dislocation managed with conservative care following early presentation and accurate identification of the injury. It is a rare injury that can be easily missed with the need for operative intervention if there is a delayed diagnosis. The dislocation was reduced and retained with an ulnar gutter splint using plaster of Paris extending from the mid-forearm to the metacarpophalangeal joints. Caution and repeated radiological assessment are advised to ensure that the reduction has been maintained. Follow-up at one week and four weeks post injury showed no pain or deformity, with a normal range of motion in the affected joints.

## Introduction

Carpometacarpal (CMC) joint dislocations are extremely rare, accounting for less than 1% of all hand injuries. They are typically linked to high-energy trauma [1]. These injuries are frequently missed because they are difficult to detect on a simple X-ray and when part of a concomitant injury [2]. CMC joint dislocations are characterized by non-specific swelling, tenderness, and crepitation over the joints. The swelling may obscure the deformity of the hand, making diagnosis difficult [3]. As a result, management could be delayed. Delay in treating CMC joint dislocations leads to poor functional outcomes and chronic residual pain [1]. Treatment is determined by the severity of the dislocation, stability of the CMC joints, and the time of hand injury management. Closed reduction is usually possible when the dislocation is recent, whereas open reduction is required for dislocations more than 15 days old [4].

## Case presentation

A male prisoner in his mid-20s presented to the emergency department with right-hand pain, swelling, and deformity after punching the wall with his closed fist. He presented about three hours after the injury. The pain was constant, with no radiation to any other part of the body, worsened with movement, slightly reduced when still, and rated 6/10 on a scale of 0 to 10. There was no associated bruising or open wounds.

He is usually fit and well, with no chronic illness or regular medications. He is right-hand dominant, smokes about 15 cigarettes daily, and has smoked for nearly 10 years with occasional alcohol intake.

On examination, it was a closed and isolated injury. There was swelling just distal to the dorsal and ulnar aspect of the right wrist, with no bruising on any part of the right hand and no distal neurological or vascular deficit. The right-hand fingers were held in a neutral position. Systemic examination showed no other injuries.

**Figure 1:**
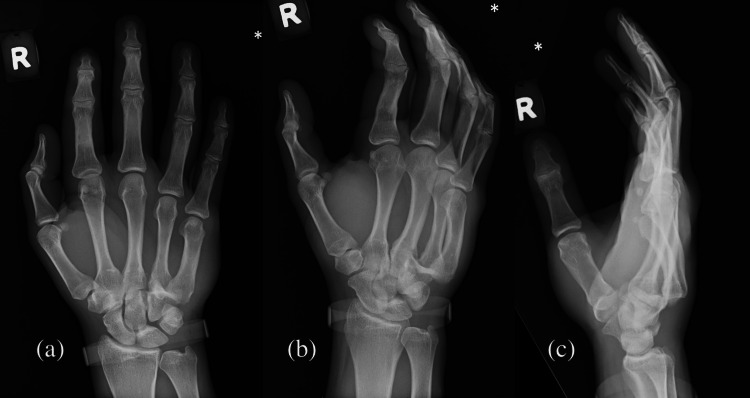
X-ray images showing dorsal dislocation of the ring and little finger carpometacarpal joints. (a) Anteroposterior view, (b) oblique view, and (c) lateral view.

He had X-rays of the right hand in anteroposterior, lateral, and oblique views (Figure 1), showing dorsal dislocation of the ring and little finger carpometacarpal joints. The attending emergency physician identified the right ring and little finger CMC joint dislocations as the diagnosis and the patient was referred to orthopaedics.

**Figure 2:**
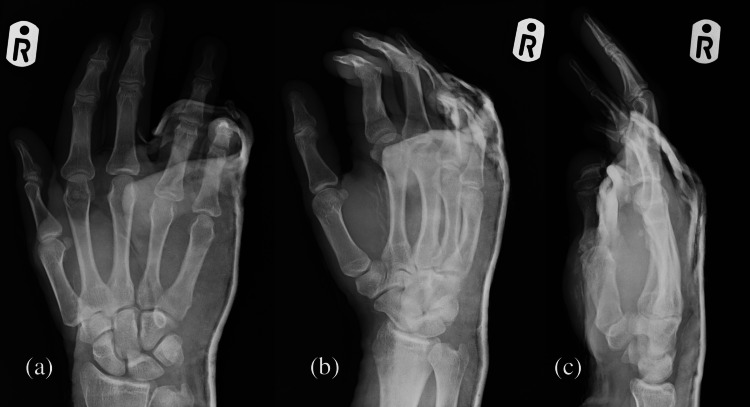
Post-manipulation check X-ray showing realignment of ring and little finger carpometacarpal joints. (a) Anteroposterior view, (b) oblique view, and (c) lateral view.

Using Penthrox (methoxyflurane) for pain relief, an assistant applied distal longitudinal traction to the affected fingers as the dislocation was pushed distally from the dorsum at the base of the metacarpals, and a discernible tactile and audible sensation was elicited, indicative of successful joint realignment. Subsequently, the reduction was retained with an ulnar gutter splint using plaster of Paris extending from the mid-forearm to the metacarpophalangeal joints, ensuring it was well moulded. The patient had a post-manipulation and cast check X-ray (Figure 2), which showed a satisfactory reduction of dislocations. He was then discharged with painkillers and advised on cast care, and the patient was to be followed up in a week.

**Figure 3:**
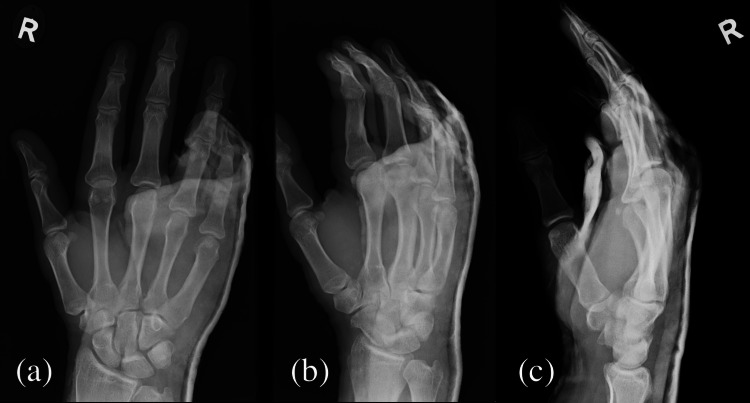
X-ray one-week post injury. (a) Anteroposterior view, (b) oblique view, and (c) lateral view.

He was examined at the fracture clinic one week after the incident. The patient was comfortable and, on examination, had no neurovascular deficit. Repeat X-rays (Figure 3) showed well-reduced ring and little finger CMC joints. He was given a complete cast and a two-week appointment for the plaster to come off and to begin the range of movement exercises.

**Figure 4:**
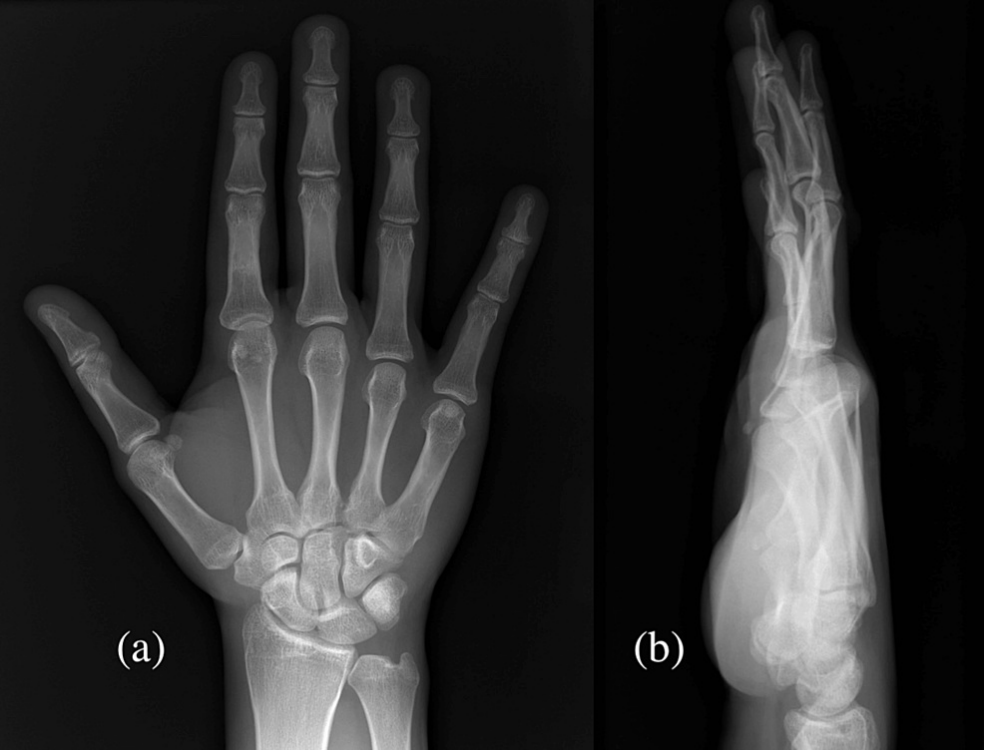
Follow-up X-ray five weeks post injury. (a) Anteroposterior view and (b) lateral view.

He was again seen at the fracture clinic four weeks after his injury. The patient removed the cast after it had been on for some time. His right wrist was neither painful nor deformed. His right fingers and CMC joints moved within a normal range. The right ring and little finger CMC joints were positioned normally on follow-up X-rays (Figure 4). He was assessed to have normal function and discharged with advice on the range of motion exercises.

## Discussion

We described the case of a young male with ring and little finger CMC joint dislocations treated conservatively.

Dislocation of the ring and little finger CMC joints without associated fractures is not a common injury, and the diagnosis may be easily missed, which can delay treatment [5-9].

These dislocations are typically fracture dislocations brought on by the avulsion of the ligaments [5]. Bony, ligamentous, and tendinous components stabilize the CMC joints. In cadaveric dissections, several differences in the surfaces and facets of the metacarpals and the row of the distal carpals have been observed. For example, the joints between the index finger metacarpal and the trapezium and those between the ring and little finger metacarpal bases are reasonably consistent, while the remaining joints have a lot of variation [10].

The unique anatomical design of the CMC joints coupled with their ligamentous attachments provides inherent stability. However, due to their extreme rigidity, only 1° to 3° of mobility is permitted at the middle and index fingers CMC joints. In contrast, because of their saddle design and weaker ligamentous attachments, the CMC joints of the ring and little fingers have higher mobility. Therefore, the ring and little finger CMC joints have a more significant risk of dislocation than the index and middle finger CMC joints, most likely due to greater mobility [11].

A sudden force in the axial direction can dislocate the ulnar CMC joints. The volar base of the ring and little finger metacarpals hit the hamate because of this axial load, which may result in a compression or avulsion fracture of the volar base of the ring finger metacarpal. In addition, the little finger metacarpal is pulled dorsally along the intermetacarpal ligaments by the ring finger metacarpal, dislocating dorsally. The hamate and capitate may sustain dorsal rim avulsion fractures because of this dislocation of the ring and little finger metacarpals [5].

For ring and little finger CMC joint dislocations, a comprehensive physical examination is required. This involves looking for visible deformities as well as palpating for discomfort or asymmetries. To identify instability, assess the range of motion of each joint. Imaging, such as CT scans and MRIs, provide comprehensive injury information in addition to X-rays. X-rays taken from various angles reveal alignment, fractures, and joint congruency [6]. CT scans give more detailed bone imaging, whereas MRIs evaluate soft tissues such as ligaments.

Treatment options for CMC dislocation injuries include open reduction with internal fixation, closed reduction with K-wire fixation, and conservative care. Although several types of treatment have been proposed, there is still no consensus on how to treat CMC dislocation injuries [9,12]. Acute CMC fracture-dislocations with inadequate reduction and delayed CMC fracture-dislocations typically necessitate open reduction [9,13].

However, as shown in our instance, an accurate diagnosis made in the acute situation and prompt care may prevent these individuals from needing surgery. If the patient is managed conservatively, careful monitoring and repeated radiological assessment are advised to ensure that the reduction has been maintained and that hand function has returned [14].

## Conclusions

In conclusion, this is an uncommon injury that can be easily missed, causing delayed treatment. As a result, patients can have poor functional outcomes and chronic residual pain. Therefore, there should be a high index of suspicion in those with trauma to the closed fist who experience constant pain that fails to settle. However, with early presentation and diagnosis by experienced clinicians, it can be managed conservatively with a gutter splint preventing the need for operative intervention and the associated risks.
